# The effect of prophylactic hemoclips on the risk of delayed post-endoscopic mucosal resection bleed for upper and lower gastrointestinal lesions: a retrospective cohort study

**DOI:** 10.1186/s12876-020-01199-x

**Published:** 2020-03-06

**Authors:** Karen Chang, Brian S. Lee, Timnit Tekeste, Andrew Nguyen, Mopelola Adeyemo, Agathon Girgis, Karl K. Kwok, H. Michael Crowson, Alicia O. Burris, Rajeev Attam, Charles T. Chaya, Theodore E. Durbin, Andrew Q. Giap, Gordon C. Hunt, John Iskander, Kevin T. Kao, Brian S. Lim

**Affiliations:** 1 Department of Internal Medicine, School of Medicine, University of California, Riverside, 900 University Avenue, Riverside, CA 92521 USA; 2 Department of Gastroenterology, Kaiser Permanente Los Angeles Medical Center, 4867 W Sunset Blvd, Los Angeles, CA 90027 USA; 3 Department of Educational Psychology, The University of Oklahoma, 820 Van Vleet Oval, Collings Hall, Room 321, Norman, OK 73019-2041 USA; 4 Department of Gastroenterology, Kaiser Permanente Downey Medical Center, 9353 Imperial Highway, Downey, CA 90242 USA; 5 Department of Gastroenterology, Kaiser Permanente Riverside Medical Center, 10800 Magnolia Avenue, Riverside, CA 92505 USA; 6 Department of Gastroenterology, Kaiser Permanente Orange County Medical Center, 3440 E La Palma Avenue, Anaheim, CA 92806 USA; 7 Department of Gastroenterology, Kaiser Permanente San Diego Medical Center, 9445 Clairemont Mesa Blvd, San Diego, CA 92123 USA

**Keywords:** Hemoclip, Endoscopic mucosal resection, Delayed bleed, Endoscopy, Colonoscopy, Polypectomy

## Abstract

**Background:**

Endoscopic mucosal resection (EMR) is a minimally invasive procedure used for the treatment of lesions in the gastrointestinal (GI) tract. There is increased usage of hemoclips during EMR for the prevention of delayed bleeding. This study aimed to evaluate the effect of hemoclips in the prevention of delayed bleeding after EMR of upper and lower GI tract lesions.

**Method:**

This is a retrospective cohort study using the Kaiser Permanente Southern California (KPSC) EMR registry. Lesions in upper and lower GI tracts that underwent EMR between January 2012 and December 2015 were analyzed. Rates of delayed bleeding were compared between the hemoclip and no-hemoclip groups. Analysis was stratified by upper GI and lower GI lesions. Lower GI group was further stratified by right and left colon. We examined the relationship between clip use and several clinically-relevant variables among the patients who exhibited delayed bleeding. Furthermore, we explored possible procedure-level and endoscopist-level characteristics that may be associated with clip usage.

**Results:**

A total of 18 out of 657 lesions (2.7%) resulted in delayed bleeding: 7 (1.1%) in hemoclip group and 11 (1.7%) in no-hemoclip group (*p* = 0.204). There was no evidence that clip use moderated the effects of the lesion size (*p* = 0.954) or lesion location (*p* = 0.997) on the likelihood of delayed bleed. In the lower GI subgroup, clip application did not alter the effect of polyp location (right versus left colon) on the likelihood of delayed bleed (*p* = 0.951). Logistic regression analyses showed that the clip use did not modify the likelihood of delayed bleeding as related to the following variables: use of aspirin/NSAIDs/anti-coagulants/anti-platelets, pathologic diagnoses (including different types of colon polypoid lesions), ablation, piecemeal resection. The total number of clips used was 901 at a minimum additional cost of $173,893.

**Conclusion:**

Prophylactic hemoclip application did not reduce delayed post-EMR bleed for upper and lower GI lesions in this retrospective study performed in a large-scale community practice setting. Routine prophylactic hemoclip application during EMR may lead to significantly higher healthcare cost without a clear clinical benefit.

## Background

Endoscopic mucosal resection (EMR) is a well-established procedure used for the treatment of superficial lesions in the gastrointestinal (GI) tract not amenable for standard resection techniques. Before the advent of EMR, surgical resection was the treatment of choice, especially for large lesions. Compared to surgical resection, EMR is minimally invasive, less costly, has a faster recovery time, and maintains the normal functions of the GI tract [1]. Moreover, EMR has a high efficacy and safety profile with an acceptably low complication profile [1–6].

Bleeding is the most common complication of EMR [7, 8]. The incidence of delayed bleeding, which can be from few days to several weeks post resection, varies from 1 to 6% [9–15]. Several risk factors have been reported to be associated with delayed bleeding: older age (≥75 years), peri-procedural anticoagulation use, American Society of Anesthesiologist (ASA) classification scores of III or IV, larger lesion size (> 40 mm), right-sided lesions and mucosal gap not closed by hemoclips [7, 16–19].

Although hemoclips are commonly utilized to prevent delayed bleeding, its application has been associated with conflicting results. While some studies have shown prophylactic clipping during EMR to be useful in preventing delayed bleed [20–22], other studies have shown no significant benefit [23–27]. In fact, upfront routine prophylactic clipping may result in improper resource utilization, including cost and time [27, 28].

There are numerous studies looking at the use of hemoclips for the prevention of post-polypectomy delayed bleeding in large colonic polyps [20, 21, 23–25], but there is a paucity of studies that examine the prophylactic use of hemoclips during EMR of upper GI lesions [22, 29]. In the present study, our aim is to analyze the efficacy of prophylactic hemoclip application to prevent post-EMR delayed bleed of both upper and lower GI lesions.

## Methods

### Study population and inclusion/exclusion criteria

The study was approved by the Kaiser Permanente Southern California (KPSC) Institutional Review Board (protocol number 10830). Kaiser Permanente Southern California is an integrated health system with 15 hospitals and 202 medical offices. Data from the KPSC EMR registry were reviewed to identify upper and lower GI lesions that underwent EMR between January 2012 and December 2015. All patients were over the age of 18. EMR procedures were performed by 12 interventional endoscopists across the region who have all undergone advanced endoscopy fellowship and routinely perform EMR as part of their practice. In addition to the primary outcome of delayed bleeding, the following variables were collected: age at diagnosis, gender, comorbidity (hypertension, diabetes, coronary artery disease, cerebrovascular accident, liver cirrhosis, and end stage renal disease), location of lesion, pathologic diagnoses, and usage of aspirin, nonsteroidal anti-inflammatory drugs (NSAIDs), anticoagulants, and antiplatelet agents. Exclusion criteria were as follows: (1) cases that were aborted due to non-lift sign, (2) aborted cases (prior to any resection) for any other reasons such as residual food and clinical instability, (3) ampullary lesions, and (4) cases where hemoclips were used for active intraprocedural bleeding and not for the sole purpose of prophylaxis.

### Definitions

Definition of prophylactic clipping post-EMR was as follows: procedures in which hemoclips were used to prevent delayed bleeding and not for the purpose of hemostasis of active intraprocedural bleeding at the time of EMR. Delayed bleed was defined as any significant GI bleeding that occurred up to 30 days post EMR. This was determined by symptoms (e.g. hematochezia and melena), hemoglobin drop of more than 1 g/dL, and/or blood transfusion(s) required. Patients were captured through a combination of hospital admissions data via KPSC electronic medical record system and claims data (for patient admissions that occurred outside of KPSC network). If there was delayed bleeding, the following variables were examined: blood transfusion(s), hospital length of stay (intensive care unit/ICU versus non-ICU), need for repeat GI procedure, IR (interventional radiology) intervention, and surgical intervention.

For those procedures that indicated clip usage but did not note specific number of clips, we assumed that at least one clip was used. There were four different models of hemoclips used for the procedures included in the study. For cost analysis of the clip expense, we used the average cost of these different models of clips which was $193.

### Statistical analysis

Statistical analysis was conducted using SPSS 24. Descriptive analyses of baseline characteristics were performed with Student’s t-test for continuous variables and chi-square test or Fisher’s exact test for binary variables. All reported *p* values had alpha 0.05 level of significance.

The primary unit of analysis in this study was lesion (level 1), which was nested within patient (level 2), who in turn was nested within endoscopist (level 3). We tested an unconditional multilevel binary logistic regression model to determine whether the likelihood of a bleed varied significantly at the level of patients and endoscopists. Results of the unconditional model revealed evidence of non-significant variation at these levels. We tested two additional two-level models – one with lesion nested within patient only and the other with lesion nested within endoscopist only. Both models yielded evidence of non-significant variation. Given this evidence of non-trivial variation at the patient and endoscopist levels (suggestive of an absence of clustering in the data), we ultimately relied on the single-level approach, i.e. at the lesion level, for statistical testing of relationships between delayed bleeding and other variables.

Fisher’s exact tests were used to test the zero-order relationship between delayed-bleed and clip use, location in the GI tract (dichotomized as upper versus lower), and size of the lesion (dichotomized as < 20 mm versus ≥20 mm). A one-sided test was utilized (under the conventional assumption that clip use would be associated with less delayed bleeding). The remaining tests involving lesion size and location variables were two-sided. Fisher’s exact test was used to evaluate the relationship between delayed bleeding and use of medications, age (dichotomized as < 75 versus ≥75), whether or not the resection was performed in piecemeal, and use of ablation. We tested the relationship between delayed bleeding and number of clips, patient age (not dichotomized), and lesion size (not dichotomized) using point-biserial correlation.

We tested whether there was a relationship between delayed bleed and final pathologic diagnoses using a chi-square test of association. Because several categories were of low frequency, we carried out our analyses involving a subset of diagnoses that exhibited frequencies of 15 or greater. As such, our analysis involved 7 of the 20 diagnostic categories found in the original data, covering 595 (91%) of the lesions.

The relationship between clip use and several additional clinically-relevant variables were examined among the 18 patients who exhibited a delayed bleed. We computed the point-biserial correlation between clip use versus no clip use and length of hospital stay. Next, we computed Fisher’s exact tests and phi-coefficients to examine the relationship between clip use and need for repeat procedures and blood transfusion requirement.

Logistic regression analyses were carried out in order to explore whether clip use (or no use) might moderate relationships between delayed bleeding and other variables (location in the GI tract [upper versus lower GI], lesion size, pathologic diagnoses, and the use of aspirin, NSAIDs, anti-coagulants, and anti-platelets). In the binary logistic regression for the lower GI subgroup, Firth procedure was used given the low incidence of delayed bleed.

Finally, we utilized multilevel binary logistic regression to test the likelihood that at least one clip would be utilized during a procedure by a particular endoscopist, as well as to explore possible procedure-level (Level 1) and endoscopist-level (Level 2) characteristics associated with clip usage during a procedure.

## Results

### Details of EMR procedures

**Fig. 1:**
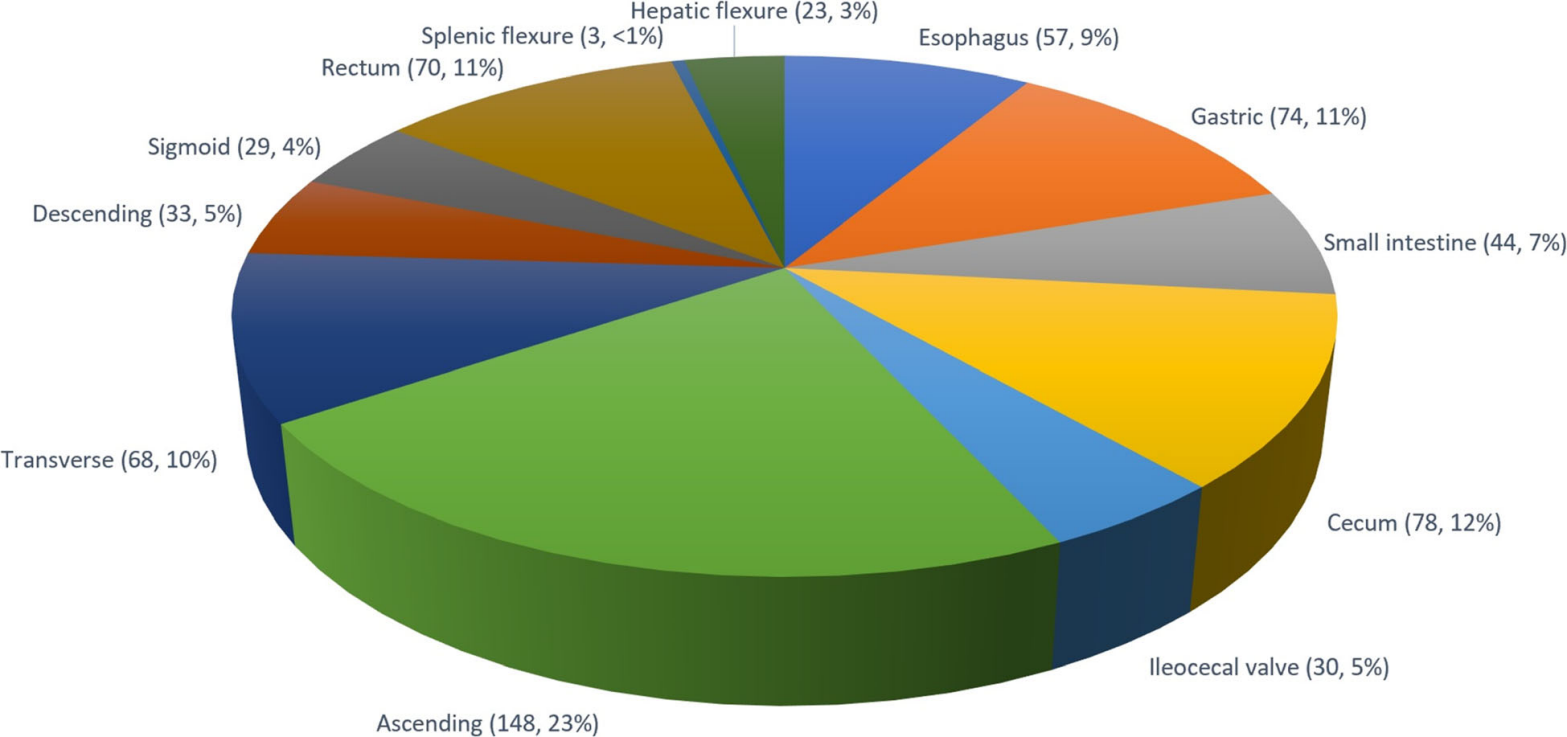
Locations of lesions detected (numbers of lesions per site, percentage)

Our analysis cohort was comprised of 620 procedures and 657 unique lesions, of which 175 (26.6%) were in the upper GI tract and 482 (73.4%) were in the lower GI tract. Twenty-nine procedures had multiple lesions (23 procedures with 2 lesions, 4 procedures with 3 lesions, 2 procedures with 4 lesions) and 591 procedures had a single lesion. The number of lesions in the prophylactic hemoclip group was 337 (51%) and 320 (49%) in the no-hemoclip group. A total of 901 clips were deployed (average of 1.37 clips per lesion). The mean size of the lesions in the hemoclip group was 23.13 mm (range 1.2–100 mm) and that of the no-hemoclip group was 27.1 mm (range 2–90 mm), *p* <  0.001. Baseline characteristics are listed in Table 1. The anatomic locations of the lesions are noted in Fig. 1.

**Table 1:** Baseline characteristics

	Clip	No Clip	*p* value
Number of lesions	337 (51%)	320 (49%)	N/A
Age [years, Mean (SD)]^b^	63.83 (10.75)	64.38 (11.43)	0.5281
Sex^b^			0.5707
Male	179 (53%)	176 (55%)	
Female	158 (47%)	144 (45%)	
Lesion size [mm, Mean (SD)]	23.13 (14.90)	27.10 (23.13)	< 0.001
Lesion location			0.0894
Upper	98 (29%)	74 (23%)	
Lower	239 (71%)	246 (77%)	
Medications^b^			
Aspirin	62 (18.3%)	56 (17.5%)	0.7903
NSAIDs	31 (9.3%)	20 (6.1%)	0.1209
Anti-platelets	6 (1.8%)	16 (5.1%)	0.0209^a^
Anti-coagulants	12 (3.6%)	9 (2.9%)	0.5968^a^
Final pathologic diagnosis			
Adenoma	186 (55.19%)	196 (61.25%)	0.1157
Hyperplastic	18 (5.34%)	10 (3.13%)	0.1598
Serrated Adenoma	37 (10.98%)	29 (9.06%)	0.4140
Adenocarcinoma	17 (5.04%)	36 (11.25%)	0.0035
Hamartoma	1 (0.30%)	0 (0.00%)	> 0.9999
Barrett’s	5 (1.48%)	10 (3.13%)	0.1592
Barrett’s Carcinoma	4 (1.19%)	5 (1.56%)	0.7466
Pancreatic Heterotopia	5 (1.48%)	1 (0.31%)	0.2175
Neuroendocrine/Carcinoid Tumor	28 (8.31%)	8 (2.50%)	0.0011
Gastric Heterotopia of Rectum	1 (0.30%)	0 (0.00%)	> 0.9999
Gastrointestinal Stromal Tumor	6 (1.78%)	1 (0.31%)	0.1239
Granular Cell Tumor	1 (0.30%)	5 (1.56%)	0.1145
Gastric Intestinal Metaplasia	5 (1.48%)	2 (0.63%)	0.4517
Leiomyoma	2 (0.59%)	1 (0.31%)	> 0.9999
Inflammatory	4 (1.19%)	2 (0.63%)	0.6867
Lipoma	6 (1.78%)	3 (0.94%)	0.5061
Squamous Cell Carcinoma	0 (0.00%)	2 (0.63%)	0.2368
Hemangioma	3 (0.89%)	0 (0.00%)	0.2494
Brunner’s Gland Hyperplasia	1 (0.30%)	1 (0.31%)	> 0.9999
Other	7 (2.08%)	8 (2.50%)	0.7168

^a^Anti-platelets and anti-coagulants are usually discontinued prior to EMR in majority of cases performed at the medical centers included although this information could not be adequately captured.

^b^Descriptive analyses for patients’ age, gender, and medication usage were performed at the patient/procedure level rather than lesion level.

### Hemoclip utilization and delayed bleeding

A total of 18 (2.7%) lesions resulted in delayed bleeding: 7 lesions (1.1%) in hemoclip group and 11 lesions (1.7%) in no-hemoclip group. Details of delayed bleeding cases are listed in Table 2. There was no statistically significant association between delayed post-EMR bleeding and use of prophylactic hemoclips, *p* = 0.204, meaning prophylactic hemoclip deployment did not reduce the delayed bleed risk. The rate of delayed bleed did not change when the analysis was stratified by upper GI (0 lesions in hemoclip group and 1 lesion in no-hemoclip group, *p* = 0.248) and lower GI (7 lesions in hemoclip group and 10 lesions in no-hemoclip group, *p* = 0.641).

**Table 2:** Detailed descriptions of delayed bleeding cases

Pt #	Location^a^	Dx^b^	Clip	Presentation			Hospitalization		Treatment			
				Days post EMR	GI Bleed symptoms	Hb decrease (g/dL)	Total LOS (days)	ICU LOS (days)	Blood transfusion (units)	GI Procedure	IR	SRG
1	6	2	Yes	5	Hematochezia	4.8	3	0	0	Colonoscopy	No	No
2	2	1	No	11	Hematochezia	5.0	4	0	0	None	No	No
3	4	1	Yes	13	Hematochezia	1.7	2	0	0	Colonoscopy	No	No
4	3	1	Yes	1	Hematochezia	3.8	4	0	4	Colonoscopy^c^,^e^	No	No
5	2	1	Yes	8	Hematochezia	2.2	2	0	0	None	No	No
6	7	4	No	0	Hematochezia	5.3	3	0	0	Colonoscopy	No	No
7	6	1	No	4	Hematochezia	4.1	3	0	0	Colonoscopy^c^	No	No
8	1	5	No	1	Melena	1.8	5	0	3	EGD^c^	No	No
9	4	1	No	9	Hematochezia	3.5	3	0	2	Colonoscopy^c^	No	No
10	3	1	No	1	Hematochezia	5.2	4	0	2	Colonoscopy ^d^	No	No
11	5	1	No	3	Hematochezia	4.2	2	0	0	Colonoscopy^c,d^	No	No
12	5	1	No	6	Hematochezia	1.7	1	0	0	None	No	No
13	2	1	Yes	3	Hematochezia	1.5	3	0	2	Colonoscopy^c^	No	No
14	4	1	Yes	13	Hematochezia	2.5	2	0	2	Colonoscopy^d^	No	No
15	4	4	No	4	Hematochezia	2.2	2	0	0	Colonoscopy	No	No
16	4	1	No	1	Hematochezia	2.4	4	0	0	None	No	No
17	4	3	No	1	Hematochezia	2.5	1	0	0	None	No	No
18	2	1	Yes	4	Hematochezia	2.8	3	0	2	Colonoscopy^c^	No	No

^a^Location code: 1-Gastric, 2-Cecum, 3-Ileocecal valve, 4-Ascending, 5-Transverse, 6-Rectum, 7-Splenic flexure.

^b^Dx (diagnosis) code: 1-Adenoma, 2-Hyperplastic, 3-Serrated adenoma, 4-Adenocarcinoma, 5-inflammatory

^c^Hemoclips applied during repeat colonoscopy/endoscopy for delayed bleed.

^d^Thermal coagulation methods applied during repeat colonoscopy/endoscopy for delayed bleed.

^e^Epinephrine injection used during repeat colonoscopy/endoscopy for delayed bleed.

*Pt* Patient, *Dx* Diagnosis, *EMR* Endoscopic mucosal resection, *Hb* Hemoglobin, *LOS* Length of stay, *ICU* Intensive care unit, *EGD* Esophagogastroduodenoscopy, *IR* Interventional radiology, *SRG* Surgery.

### Relationships between delayed bleeding and the remaining variables besides hemoclip application

There was a significant association between delayed bleeding and lesion size (< 20 mm versus ≥20 mm, *p* = 0.045) and near significance between delayed bleeding and lesion location (*p* = 0.054). Delayed bleeding occurred more frequently in those cases involving larger lesions and in lower GI. When the lesion size was treated as a continuous variable (not dichotomized as < 20 mm versus ≥20 mm), there was still a significant association between delayed bleeding and size [r(655) = 0.09, *p* = 0.02]. Of the tests involving the various medications (aspirin, NSAIDs, anti-platelets, and anti-coagulants), only anti-coagulant use was significantly related to delayed bleed (*p* = 0.017). Procedures where ablative methods were used during EMR were associated with delayed bleeding (*p* = 0.036). There was no statistically significant relationship between delayed bleed and any of the following variables: underlying conditions (hypertension, diabetes, coronary artery disease, cerebrovascular accident, cirrhosis, end-stage renal disease), age (< 75 versus ≥75), final pathologic diagnoses, different types of colon polypoid lesions (adenoma, hyperplastic, serrated adenoma, adenocarcinoma), and piecemeal resections (Table 3). Patients’ age, when treated as a continuous variable and not dichotomized, was still not correlated with delayed bleeding [r(655) = 0.078, *p* = 0.278]. Likewise, there was no correlation between delayed bleed and the number of clips used on a lesion [r(655) = − 0.021, *p* = 0.592].

**Table 3:** Fisher’s *p*-values and reported phi-coefficients associated with tests of the zero-order relationships between delayed bleeding and the variables

Variable	Fisher’s exact *p*-value	phi-coefficient (Φ)
Clip use	0.204 (one-sided)	−0.042
Lesion size (< 20 mm versus ≥20 mm)	0.045	0.079
Lesion location (upper versus lower GI)	0.054	−0.080
Aspirin Use	1.00	−0.005
NSAID use	1.00	−0.013
Coagulant use	0.017	0.129
Anti-platelet use	0.463	0.021
HTN	1.00	0.003
DM	1.00	−0.002
CAD	0.416	0.036
CVA	1.00	−0.030
Cirrhosis	1.00	−0.025
ESRD	0.222	0.060
Age at diagnosis (< 75 versus ≥75)	0.346	0.042
Ablation	0.036	0.464
Piecemeal resection	0.093	0.018
Adenoma (colon)	1.000	0.011
Hyperplastic (colon)	0.388	0.036
Serrated adenoma (colon)	0.488	−0.046
Adenocarcinoma (colon)	0.231	0.055

### Logistic regressions predicting likelihood of delayed bleed associated with clinical and procedure-related features

The first logistic regression model included lesion location (upper versus lower GI) and lesion size (mean centered) as predictors of delayed bleeding, along with two interaction terms (i.e. clip × lesion size; clip × location). There was no evidence that clip use (versus no clip use) moderated the effects of lesion size (p = 0.954) or lesion location (*p* = 0.997) on the likelihood of a delayed bleed. Furthermore, a binary logistic regression in the lower GI subgroup to test whether clip use might serve as a moderator of any relationship between polyp location (right versus left colon) on the likelihood of delayed bleed showed that the effects of clip use, polyp location in the colon, and the interaction between these variables as predictors of delayed bleed were not statistically significant [χ^2^(3) = 0.34, *p* = 0.951]. Binary logistic regressions for colon polypoid lesions (adenoma, hyperplastic, serrated adenoma, adenocarcinoma) to test whether clip use might moderate any possible effects of lesion type on delayed bleed showed that none of these models were statistically significant as follows: clip use and adenoma (*p* = 0.755), clip use and hyperplastic polyps (*p* = 0.370), clip use and serrated adenoma (*p* = 0.765), clip use and adenocarcinoma (*p* = 0.347).

Logistic regression analyses also examined whether clip use modified the likelihood of delayed bleeding as related to the following variables: use of aspirin, NSAIDs, anti-coagulants, and anti-platelets (Model 1), pathologic diagnoses (Model 2), and whether ablation and piecemeal approach was used during a procedure (Model 3). None of the predictors in the models were statistically significant (model 1 *p-*value = 0.348, model 2 p-value = 0.562, model 3 p-value = 0.064).

### Exploratory analysis: predicting use of clips during procedures

An exploratory analysis was used to predict the use of clips during procedures by the different endoscopists. Multilevel analysis showed that there is evidence of variation among endoscopists in their likelihood to use at least one clip during a procedure. Nevertheless, the candidate predictors (average age of patients, the number of previous procedures, and the average size and number of lesions across an endoscopists’ procedures) were unrelated to clip use. In short, there are probably other undetermined factors associated with the endoscopists’ decision tree that might account for the variable likelihood of using clips.

### Cost analysis

Given that the total number of clips was 901 and the average cost of different clips used was $193, the total cost of clips for prophylactic purposes was $173,893. The actual additional cost incurred by clip usage is probably higher given that procedures that did not specify the number of clips were analyzed as having used 1 clip and the true clip number is likely to be higher. Since there was no statistically significant difference between clip use and several clinically-relevant variables (that have the potential to accrue additional medical cost) among the 18 patients who exhibited delayed bleeding (hospital length of stay, *p* = 0.725; need for repeat procedure at the time of rebleed, *p* = 0.596; and blood transfusion requirement, *p* = 0.332), it can be assumed that $173,893 is the majority of the direct costs of prophylactic clip usage, not including the indirect costs such as prolonged procedure time. None of the delayed bleeding cases required ICU admission, IR intervention, or surgery.

## Discussion

The practice of prophylactic application of hemoclips for the prevention of delayed bleeding is increasing [30]. However, placement of hemoclips has been associated with mixed results in preventing delayed EMR bleeding and there are no uniform guidelines to support its routine application for this purpose. Furthermore, the cost of hemoclips are rather substantial with price range of $ 125–277 per clip in the current market (average cost for the four different models of clips used for this study cohort was $ 193), and this does not include added time spent to perform clip application thus raising concerns about the cost-effectiveness of routine prophylactic clip placement [25, 31]. Parikh et al. searched existing published data on post-polypectomy bleeding rates and performed a decision analysis to examine the cost-effectiveness of routine, prophylactic clip placement after colon polypectomy [31]. The researchers determined that prophylactic clip placement seems to be a cost-effective strategy in patients who are taking anti-platelet or anti-coagulation medications at the time of the procedure. However, for those patients not receiving anti-platelet or anti-coagulation therapy, placement of prophylactic clips after polypectomy was not cost-effective, particularly for smaller sized polyps [31]. Dokoshi et al. conducted a randomized study on the effectiveness of prophylactic clipping during endoscopic resection of colon polyps for the prevention of delayed bleeding [26]. They concluded that there was no difference in delayed bleeding rates between patients who received prophylactic clipping and those who did not. In this particular study, anticoagulant treatment made no difference between the clip and non-clip group in delayed bleeding rates provided the anticoagulant was stopped prior to the procedure [26]. Mori et al. carried out a prospective trial comparing snare cauterization to clip closure for the prevention of post-EMR delayed bleed. They concluded that snare cauterization was superior to clip closure in terms of procedure time and medical costs. Moreover, their findings showed no difference in delayed bleeding between the two groups [27].

Our study is a large retrospective cohort study that examines the effect of hemoclip application in preventing delayed post-EMR bleed for both upper and lower GI tract lesions. There was no statistically significant association between delayed post-EMR bleeding and use of prophylactic hemoclips. The total cost of clips for prophylactic purposes was $173,893 while there was no statistically significant difference between clip use and several clinically-relevant variables (hospital length of stay, need for repeat procedure at the time of rebleed, and blood transfusion requirement) that have the potential to accrue additional medical cost, meaning the amount listed here was the majority of the direct cost of prophylactic clip usage. Given these findings, one can then question if there is no place for prophylactic hemoclip placement at all in the setting of upper/lower EMR or certain subgroups within these EMR procedures. When we performed analyses on delayed bleeding rate in the context of different variables without taking clipping into consideration, delayed bleed did occur more frequently in those cases involving larger lesions (whether the lesion size was treated as a continuous variable or dichotomized as < 20 mm versus ≥20 mm), lower GI lesions, anti-coagulant use and ablative method usage during EMR. When logistic regression was performed, however, we found that there was no evidence that clip use (versus no clip) moderated the effects of lesion size, lesion location (upper versus lower), anti-coagulation use, usage of ablation, colon polyp location (right versus left) on the likelihood of delayed bleed. Therefore, one can perhaps suspect that prophylactic clipping may have a role in high-risk subgroups (larger lesions, lower GI lesions, anti-coagulation use, and ablation at the time of EMR) based on their higher rate of delayed bleeding, but our study did *not* show that hemoclip application in these cohorts resulted in lower delayed bleed.

The primary limitation of this study is its retrospective design, which did not allow ideal variable control between the two groups. Nonetheless, our secondary analyses addressing these variables did not change the overall result of the study. Another limitation is that the procedures were performed by multiple endoscopists with differences in training and technique. However, our post-hoc analysis showed that the inter-endoscopist factor does not change the primary outcome. It may be argued that the endoscopists used hemoclips primarily for the lesions that had increased of risk of bleeding. This type of information, i.e. endoscopist bias, would be hard to capture in a retrospective study. While it is possible that there may have been some bias toward using clips for those cases that the endoscopists thought had higher chance of bleeding based on lesion characteristics and/or patient characteristics, following reasons confirm that these biases were minimal. In terms of lesion characteristics, one information our study captured is the lesion size. We noted that those with no clips had larger lesion size (mean 27.10 mm) compared to those with clips (mean 23.13 mm), suggesting that the endoscopists did not necessarily feel compelled to use hemoclips for larger size lesions. For patient characteristics, there was no difference between clip versus no-clip groups in terms of gender, age, aspirin use, NSAID use, and anti-coagulation use. There was a difference in anti-platelet usage between two groups, but the no-clip group had a higher proportion of patients who used anti-platelets, meaning the endoscopists did not feel obliged to use clips more often in this cohort solely because they were on anti-platelets. Lastly, the number of clips used was unclear in some of the EMR procedures and thus the cost analysis was an estimation. However, given that we did not overestimate the number of clips in those unclear cases but instead underestimated the number of clips used, i.e. performed analyses of these “unclear” procedures as having only one clip, the actual additional cost incurred by clip usage is likely to be higher.

Our present study has several strengths as well. The integrated healthcare system of KPSC allows for a central database repository that yielded a large sample size and comprehensive data availability during the follow-up period after EMR. The Kaiser Permanente patient population is a rather diverse population of different geographical and ethnic backgrounds, which supports these study results being more generalizable. Lastly, our data includes both upper and lower gastrointestinal tract lesions whereas the majority of the studies that have examined the prophylactic use of hemoclips for EMR were restricted to the lower GI system.

There is a paucity of large, randomized controlled trials (RCT) that specifically examine the cost-effectiveness of prophylactic hemoclip utilization for *both* upper and lower EMR, but several recent RCTs, published after the completion of our study, on the usage of hemoclips to prevent delayed bleeding (DB) after resection of colon lesions may be worth mentioning. First of these studies was by Pohl et al. examining large nonpedunculated colon polyp (≥ 20 mm) that underwent endoscopic closure with a clip or no closure [32]. The protective effect of clip closure of the mucosal defect in reducing the risk of DB appeared to be restricted to the proximal colon lesions; there was actually higher delayed bleeding in distal colon lesions in the clip group. Subsequently, a study by Albeniz et al. also included nonpedunculated colon polyps measuring ≥20 mm but with substantial DB risk, i.e. > 90% of the cases were proximal polyps, 51% were > 40 mm, and 36% of the patients were on antiplatelets [33]. Intention to treat (ITT) analysis showed a trend for lower DB in clip group. In per protocol (PP) analysis, DB was significantly lower in complete clip closure group (as opposed to the partial closure subgroup and the failed closure subgroup). However, these polyps that were successfully clipped were also smaller, had better accessibility, and in shorter and easier EMR procedures. Contrary to the result of these two studies mentioned, the study by Feagins et al. showed no statistical difference in BD between hemoclip group and no hemoclip group in their cohort of 1098 patients [30]. As opposed to the Pohl study, they found no difference in bleed rates between proximal polyps that underwent prophylactic hemoclip placement versus no prophylactic hemoclip. While these recent RCTs have overcome one of the limitations of our study, its retrospective design, our study still has an advantage since it includes both upper GI and lower GI/colon lesions. Also, unlike the study by Albeniz et al., which included only high-risk lesions, our study included all lesions regardless of their risk status, and this may potentially make our study more generalizable for all EMRs. The Feagins study supports the findings of our results that the prophylactic placement of hemoclips does not affect the proportion of DB, also questioning the widespread, expensive practice of routinely placing prophylactic hemoclips after polyp resection.

## Conclusion

In conclusion, prophylactic hemoclip application did not appear to result in decreased delayed post-EMR bleed for upper and lower GI tract lesions in this retrospective analysis. Given this result and the cost of hemoclips, routine prophylactic clip application during EMR may incur high healthcare costs without consistently demonstrable clinical gains. The practice of healthcare is constantly changing to incorporate more cost-effective care, and thus the outcomes of this study have important implications.

## Data Availability

The datasets used and/or analysed during the current study are available from the corresponding author on reasonable request.

## Abbreviations

ASA: American Society of Anesthesiologist
CAD: Coronary artery disease
CVA: Cerebrovascular accident
DB: Delayed bleeding
DM: Diabetes
EGD: Esophagogastroduodenoscopy
EMR: Endoscopic mucosal resection
ESCA: post-EMR scar clip artifact
ESRD: End stage renal disease
GI: Gastrointestinal
Hb: Hemoglobin
HTN: Hypertension
ICU: Intensive Care Unit
IR: Interventional radiology
KPSC: Kaiser Permanente Southern California
LOS: Length of stay
NSAIDs: Non-steroidal anti-inflammatory drugs
RCT: Randomized controlled trial
